# Important functions and molecular mechanisms of aquaporins family on respiratory diseases: potential translational values

**DOI:** 10.7150/jca.98829

**Published:** 2024-10-07

**Authors:** Jinshan Li, Dongyong Yang, Lanlan Lin, Liying Yu, Luyang Chen, Kaiqiang Lu, Jieli Lan, Yiming Zeng, Yuan Xu

**Affiliations:** 1 Department of Pulmonary and Critical Care Medicine, The Second Affiliated Hospital of Fujian Medical University, Quanzhou, Fujian Province, 362000, China.; 2 Fujian Provincial Key Laboratory of Lung Stem Cells, Quanzhou, Fujian Province, 362000, China.; 3 Fujian Provincial Clinical Research Center of Interventional Respirology, Quanzhou, Fujian Province, 362000, China.; 4 Central Laboratory, The Second Affiliated Hospital of Fujian Medical University, Quanzhou, Fujian Province, 362000, China.; 5 Clinical Research Unit, The Second Affiliated Hospital, Fujian Medical University, Quanzhou, China.; 6 School of Public Health, Fujian Medical University, Fuzhou, Fujian Province, 350000, China.

**Keywords:** aquaporins, chronic obstructive pulmonary disease, bronchial asthma, acute respiratory distress syndrome, non-small cell lung cancer

## Abstract

Aquaporins (AQPs) are a subgroup of small transmembrane transporters that are distributed in various types of tissues, including the lung, kidney, heart and central nervous system. It is evident that respiratory diseases represent a significant global health concern, with a considerable number of deaths occurring worldwide. Recent researches have demonstrated that AQPs play a pivotal role in respiratory diseases, including chronic obstructive pulmonary disease (COPD), asthma, acute respiratory distress syndrome (ARDS), and particularly non-small cell lung cancer (NSCLC). In the context of NSCLC, the overexpression of AQP1, AQP3, AQP4, and AQP5 has been demonstrated to facilitate tumor angiogenesis, as well as the proliferation, migration, and invasiveness of tumor cells. This review concisely explores the role of AQP family on respiratory diseases, to assess their clinical and translational significance for understanding molecular pathogenesis. However, the potential translation of AQPs biomarkers into clinical applications is promising and the understanding of the precise mechanisms influencing respiratory diseases is still ongoing. Addressing the challenges and outlining the future perspectives in AQPs development is essential for clinical progress in a concise manner.

## Introduction

Aquaporins (AQPs) are multifunctional transmembrane channel proteins that play a crucial role in regulating membrane water permeability with phosphorylation, pH, Ca^2+^, and osmotic pressure gradients^1^. In addition, the AQPs retain their ability to promote the transport of gases, ions and signaling factors^2^. AQP1 was first isolated from the erythrocyte membrane as a 28-kDa bilayer-spanning polypeptide. Subsequently, other members of the AQPs family were identified^3^. The AQP family comprises a comprehensive set of 13 members (AQP0 to AQP12), providing valuable insights into the fundamental physiology of the regulation of water balance in vital organs, such as the lung, kidney and heart^4^.

**Figure 1:**
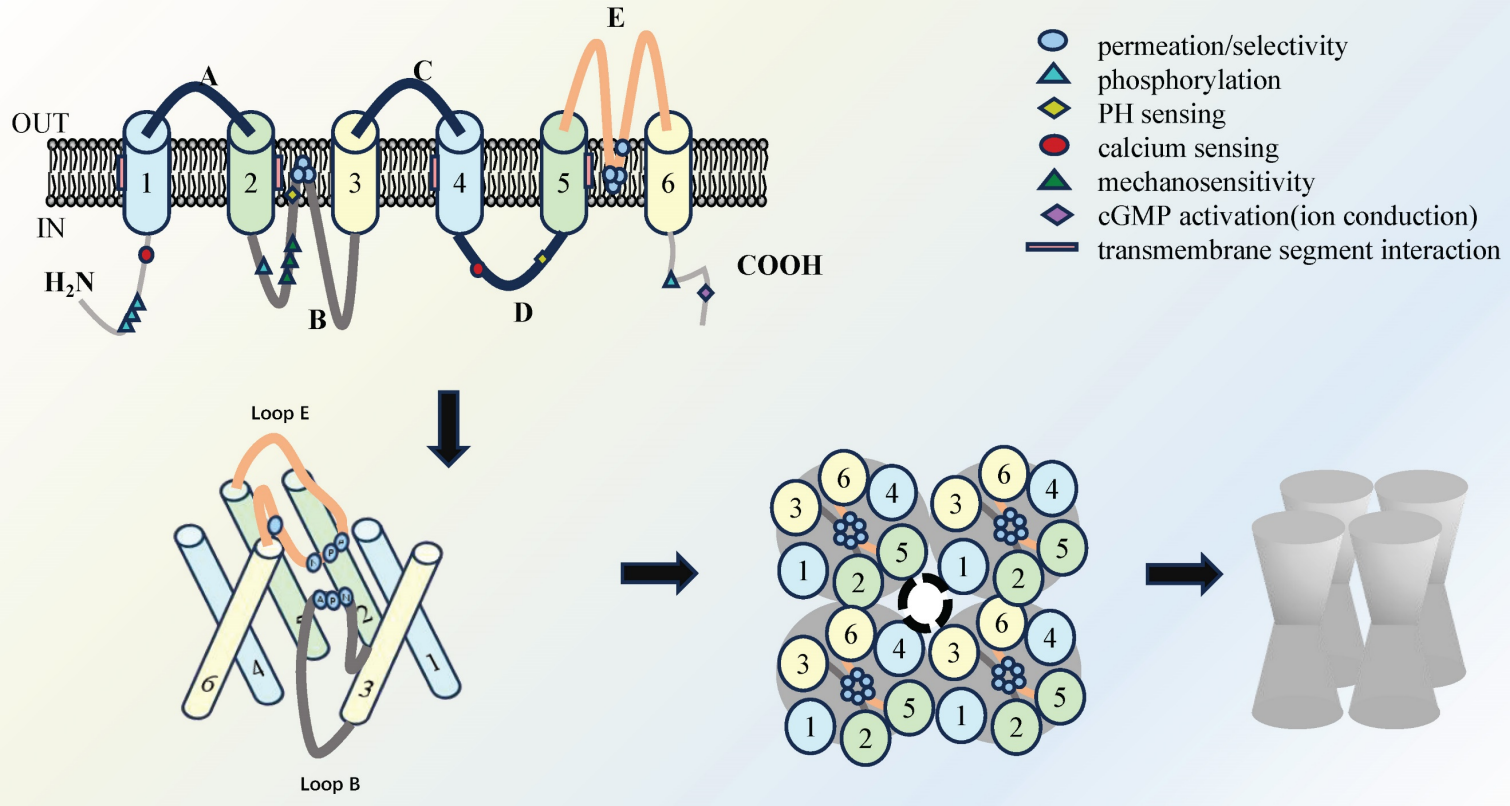
Schematic overview of the molecular structure of AQP: Four monomers that cross the cell membrane six times make up an "hourglass" tetramer compound AQPs, each monomer has a single functional pore, and the four monomers also form a central pore.

The recognized features of AQPs involve a tetramer structure, where each monomer spans the cell membrane six times, creating an "hourglass" configuration. In addition, each monomer contributes to a functional pore and collectively form a central pore, which is determined by the diameter of the pore and the intrinsic binding site, and thus determines the selectivity for water molecules (**Figure 1**)^5^. The AQPs family are traditionally categorized into three primary groups based on amino acid sequence homology and permeability characteristics. The classical AQPs group is identified in higher mammals and encompasses AQP0, AQP1, AQP2, AQP4, AQP5, AQP6, and AQP8^6^. In recent years, aquaglyceroporins, including AQP3, AQP7, AQP9, and AQP10, have been identified and demonstrated to permit the passage of small uncharged solutes. Of note, AQP11 and AQP12 are classified as subcellular aquaporins and exhibit low sequence homology with other AQPs^7^.

**Figure 2:**
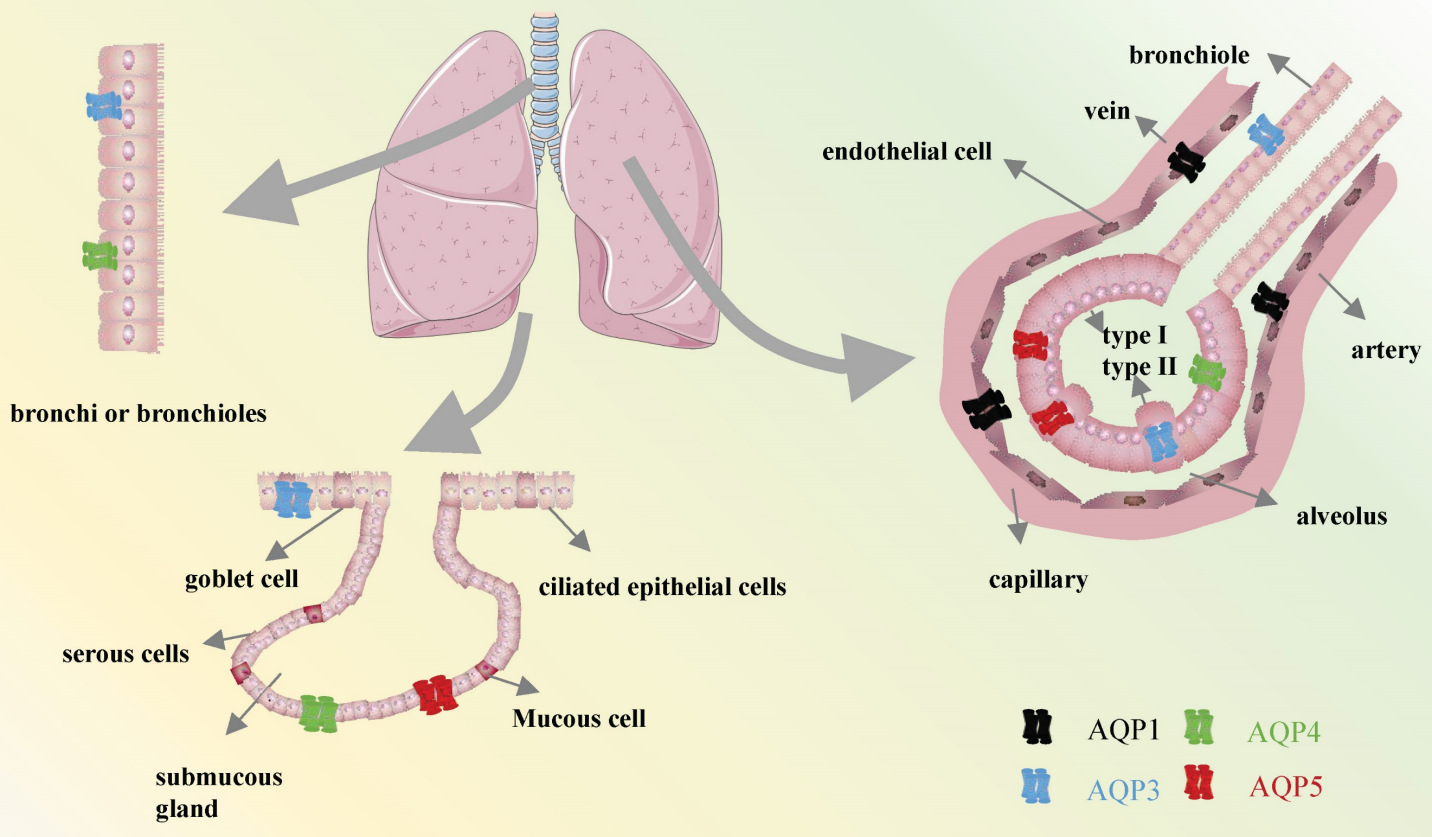
Distribution of AQPs in the respiratory system.

Among the identified members, the expressions of AQP1, AQP3, AQP4, and AQP5 have been observed in various cell types, where they have been shown to contribute to the maintenance of pulmonary function homeostasis^4^, ^8^, ^9^. It is of the utmost importance to expel fluid from the lung in order to facilitate the entry of air into the lungs and the initiation of spontaneous breathing at birth. This process is largely dependent on the capacity of AQPs within epithelial cells to effectively absorb large quantities of water^10^, ^11^. Previously, AQP1 was reported to be observed in the endothelial cells lining pulmonary blood vessels, as well as in both the apical and basolateral membranes of the pleural microvascular endothelium^12^. AQP3 was identified in the endothelial cells lining pulmonary blood vessels, as well as in the apical and basolateral membranes of the pleural microvascular endothelium^13^. AQP4 has an essential impact on the outer membrane of bronchial and tubular columnar cells in mice, along with in type 1 alveolar epithelial cells^14^. In addition, AQP5 is also expressed in the apical membrane of type 1 alveolar epithelial cells and submucosal glandular serous cells of the upper bronchus, as well as in type 2 alveolar cells in mice (**Figure 2**)^15^. Given the distribution of AQPs members in different cell types in lung tissues, they are likely to be used as key elements in respiratory diseases. This review focuses exclusively on the molecular function and specific mechanisms of AQPs to identify potential therapeutic strategies for respiratory diseases.

## AQPs and chronic obstructive pulmonary disease

### Mucus secretion and AQP5

A common manifestation of Chronic Obstructive Pulmonary Disease (COPD) is the persistent presence of cough and sputum production^16^, ^17^. As a matter of fact, mucus is mainly composed of water, which makes up about 95% of its composition, together with salts, lipids, proteins, and glycoproteins^18^. Excessive mucus synthesis and secretion are the primary causes for sputum production, impaired ciliary clearance and gas exchange^19^. This condition may give rise to abnormal bacterial colonization and airway obstruction^20^. Therefore, targeting mucus secretion is emerging as a therapeutic avenue for symptom control in COPD.

In previous studies, AQP5 expression was found to be reduced in the airways of COPD patients with mucus hypersecretion, which might correlate with the severity of airway obstruction (**Table 1**)^21^, ^22^. However, more research is needed to elucidate the underlying mechanisms. Genetic variants of AQP5 have been implicated in the rate of decline in pulmonary function, as observed in studies that identified five single nucleotide polymorphisms (SNPs) on 12q13 and two additional SNPs on the AQP5 gene^22^. Among these SNPs, the noteworthy variant rs3736309, located in intron 3, has a reduced prevalence of COPD with carriers of the G allele in the Chinese population^22^, ^23^. The findings suggest that rs3736309 is a risk locus for COPD. Targeting AQP5 expression and genetic regulation may represent promising therapeutic strategies for the management of COPD.

### TNF-α-AQPs-MUC5AC regulating shaft and inflammatory progression

**Figure 3:**
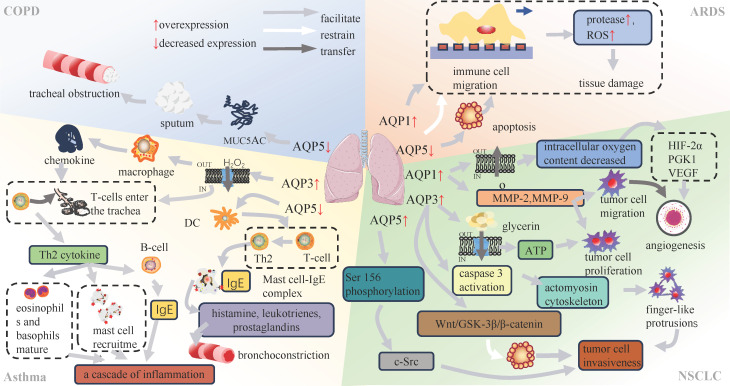
Association of AQPs with respiratory diseases.

Mucin 5AC (MUC5AC) is a marker of mucus production of airway epithelium^24^, ^25^. In previous studies, a kind of COPD model was induced by lipopolysaccharide (LPS) stimulation of human airway submucosal cells (SPC-A1 cells). The results demonstrated a dose-dependent reduction in MUC5AC secretion and an increase in AQP5 expression (**Table 1**)^26^, suggesting that both AQP5 and MUC5AC were involved in the inflammatory processes in lung tissue. Furthermore, AQP5 showed a negative correlation with inflammatory mediators, including tumor necrosis factor (TNF)-α and interleukin (IL)-6^27^. In contrast, MUC5AC showed a positive association with the progression of lung inflammation through the nuclear factor κB (NF-κB) and IL-13-STAT6-SAM pathways (**Figure 3**)^28^, ^29^. The TNF-α-AQPs-MUC5AC Regulating Shaft (TAMR) is closely associated with the inflammatory progression of COPD, yet the specific mechanism governing the interplay between AQP5 and MUC5AC in this regulatory axis remains unclear^30^. Nevertheless, elucidation of the interaction between AQPs and COPD remains imperative and offers a potential avenue for identifying therapeutic targets in the context of these diseases.

## AQPs and asthma

### AQP3 in chronic airway inflammation

Bronchial asthma, commonly known as asthma, is a chronic inflammatory disorder of the respiratory system. It is characterized by persistent airway inflammation, heightened airway responsiveness, excessive mucus production and structural changes in the airways^31^, ^32^. The main determinant of chronic airway inflammation and airway hyperresponsiveness is the accumulation of inflammatory cells within a subepithelial layer by cellular migration^33^. Studies have shown an elevation in both AQP3 mRNA and protein expression levels induced by both the allergen ovalbumin (OVA) and interleukin-13 (IL-13) in an asthma mouse model (**Table 2**)^34^. AQP3, which belongs to the second class of aqua-glyceroporins, is involved in facilitating membrane uptake of hydrogen peroxide (H_2_O_2_), a specific type of reactive oxygen species (ROS)^35^, ^36^. This process modulates chemokine production by alveolar macrophages and affects T-cell trafficking (**Figure 3**)^35^, ^37^. T cells that enter the airways are responsible for coordinating the inflammatory response by secreting cytokines and other mediators^38^. T helper type 2 (Th2) cytokines, in particular interleukin IL-4 and IL-13, are involved in the class switching of B cells for the synthesis of immunoglobulin E (IgE), the recruitment of mast cells, and the maturation of eosinophils and basophils^35^, ^39^.

*In vivo*, mice that received ovalbumin (OVA)-sensitized spleen cells from AQP3 knockout (AQP3^-/-^) mice exhibited diminished airway eosinophilic inflammation. Additionally, there was a reduced migration of CD4+ T cells to the lungs in AQP3^-/-^ mice, ultimately resulting in significantly reduced airway inflammation compared to wild-type mice^35^. It is therefore proposed that the up-regulation of AQP3 expression is intricately linked to the accumulation of inflammatory cells in asthma.

### AQP5 in airway hyperresponsiveness

While airway hyperresponsiveness is typically attributed to immune responses and inflammatory factors causing damage to mucosal epithelial cells, leading to exposure of airway nerve terminals, there is also a belief that airway hyperresponsiveness is associated with the diminished expression of AQP5^40^-^43^. It has been demonstrated that AQP5 knockout (AQP5^-/-^) mice exhibit heightened responsiveness to bronchoconstriction induced by cholinergic stimulation^22^. Previous studies have demonstrated that elevated levels of AQP5 expression are observed on the surface of bone marrow-derived dendritic cells (mDC) in both *in vivo* and *in vitro* settings (**Table 2**). Furthermore, the expression of CD80 and CD86, along with the intracellular phagocytic capacity of immature mDC in AQP5^-/-^, was found to be lower than that in wild-type mice. However, these differences were nullified after LPS induction^44^. The collective findings indicate that AQP5-mediated water transmembrane processes may be involved in the functioning of dendritic cells (DCs).

In the interim between antigen capture and the subsequent presentation of antigens to T cells, dendritic cells migrate to lymph nodes, where they initiate the stimulation of T cells through the major histocompatibility complex (MHC)-peptide complex^45^, ^46^. DCs are responsible for the differentiation of CD4+ T cells into either interferon (IFN)-γ-producing T helper type 1 (Th1) cells or interleukin (IL)-4-producing Th2 cells^44^, ^47^. Th2-mediated inflammation is facilitated by the involvement of Th2 cells in the production of immunoglobulin E, which in turn binds to mast cell receptors, initiating the formation of complexes between these two cell types (**Figure 3**). In response, these complexes are able to recognize allergens and initiate the secretion of histamine, leukotrienes, and prostaglandins^48^. Consequently, the activation of airway smooth muscle occurs, resulting in bronchial constriction and airway hyperresponsiveness^49^. It is a reasonable hypothesis that the downregulation of AQP5 is closely linked to airway hyperresponsiveness.

### AQPs levels and sputum MUC5AC

AQP5 plays a similar role in both asthma and COPD, as its downregulation may contribute to increased mucus secretion in individuals with asthma^50^. In the study, house dust mite (HDM)-induced asthma mice displayed features of airway inflammation, Th2 cell aggregation, and mucin hypersecretion comparable to those observed in AQP5 gene knockout mice. Importantly, a significant reduction in the protein and gene expression levels of MUC5AC and MUC5B was noted in the lung tissue of AQP5 gene knockout mice^51^, ^52^. This substantiates the implication of AQP5 in the progression of chronic inflammation and mucus hyperplasia in the context of asthma.

Furthermore, it implies that AQP1 is implicated in mucus hypersecretion, and the expression patterns of AQP1 and AQP5 in small airways and alveoli exhibit divergent trends^53^. The upregulations of AQP1 and AQP5 expression in small airways suggests their potential involvement in the development of submucosal edema and excessive mucus production (**Table 2**)^53^. On the contrary, diminished levels of AQP1 and AQP5 expression in the alveoli may contribute to heightened viscosity of alveolar fluid and the formation of mucus plugs^53^. Hence, our hypothesis posits that both AQP1 and AQP5 could serve as potential markers for asthma. Upon analyzing sputum samples from 34 patients with mild to moderate asthma, a positive correlation was observed between AQP1 and AQP5 levels. Additionally, a positive correlation was noted between sputum MUC5AC and both AQP5 and AQP1^54^. Nevertheless, the assessment of AQPs expression in patients with severe asthma was precluded due to the contraindications for induced sputum. In consideration of these findings, it can be inferred that AQP1 and AQP5 might be as dependable markers for evaluating mild to moderate asthma^54^.

### AQP4 in asthmatic BECs and lung fluid regulation

The upregulation of AQP4 expression was detected in bronchial epithelial cells (BECs) among individuals with asthma (**Table 2**)^55^. The associations between pulmonary edema and airflow obstruction, as well as airway responsiveness, have been substantiated in patients with asthma^56^. Building upon these pieces of evidence, we conjecture that the upregulation of AQP4 expression in bronchial epithelial cells of asthmatic patients might constitute one of the mechanisms employed by the lungs to efficiently eliminate excess fluid.

### Plant extracts in asthma treatment via modulating AQPs expression

The impact of dexamethasone, ambroxol, and terbutaline on the expression of AQP mRNA and protein in OVA-induced asthma mice can be elucidated. After24 hours of the last OVA exposure, the mRNA expression levels of AQP1, AQP4, and AQP5 showed a significant decrease, whereas AQP3 expression exhibited a notable increase^57^. At the protein level, it presented decreased AQP1 and AQP5 expression and increased AQP3 expression, with no observable changes in AQP4. Correspondingly, following the administration of the three anti-asthmatic drugs, despite a reduction in pulmonary edema, there was an increase in the mRNA and protein expression of AQP1 and AQP5. Moreover, dexamethasone and ambroxol not only upregulated the expression of AQP1 and AQP5 but also demonstrated potent anti-inflammatory effects, safeguarding the alveolar-capillary barrier. In contrast, terbutaline solely upregulated the expression of AQP1 and AQP5^57^.

Furthermore, there are ongoing initiatives to explore the potential of substituting traditional herbal medicine with alternative options. Quercus leucotrichophora extract^49^, ziziphora clinopodioides^58^, teucrium stocksianum^59^, curcumin^60^ and pistacia integerrima^53^ have all been shown to inhibit the expression of proinflammatory cytokines to improve airway inflammation, increase the expression levels of AQP1 and AQP5 to reduce pulmonary edema, and thus improve asthma symptoms. Thus, AQPs play a pivotal role in the pathogenesis of asthma and represent a potential therapeutic target. In the future, further exploration is warranted in the realm of herbal medicine for asthma treatment, particularly in the context of regulating AQPs.

## AQPs and acute respiratory distress syndrome

### Limited role of AQPs in pulmonary edema

The principal pathological features of acute respiratory distress syndrome (ARDS) entail inflammation-induced injury to the pulmonary microvascular endothelium and alveolar epithelium. This leads to the heightened permeability of pulmonary microvasculature, the efflux of protein-rich fluid from the alveolar cavity, and the subsequent onset of pulmonary edema, along with the formation of hyaline membranes^61^, ^62^. Physiologically, alveolar epithelial cells regulate fluid balance by creating osmotic gradients through the utilization of various proton pumps and channels^63^, ^64^. This establishes an equilibrium wherein fluid transport into the intercellular matrix from the blood is balanced by reabsorption into the lymphatic vessels^65^, ^66^. The emergence of interstitial edema is ascribed to an increase in capillary pressure, resulting in an excess of fluid clearance^67^, ^68^. The tight epithelial barrier serves to shield the alveolar space from the formation of edema^69^. Nonetheless, when the epithelial barrier is compromised, protein permeability rises, causing the loss of the osmotic gradient. Consequently, interstitial fluid enters the alveolar space through both AQPs and non-AQPs channels, culminating in the development of pulmonary edema^61^.

AQP1 and AQP5 are primarily expressed in capillary endothelial cells and alveolar epithelial cells, facilitating the transport of water (**Table 3**)^70^. However, prior studies have shown that the absence of AQP1 and AQP5 does not significantly influence the formation and absorption of pulmonary edema, suggesting a restricted association between AQPs and pulmonary edema^71^. The slower rate of fluid transport through AQPs, in comparison to cellular bypass, may account for the observed lack of influence of AQP1 absence.

### Inflammation and immune response

**Figure 4:**
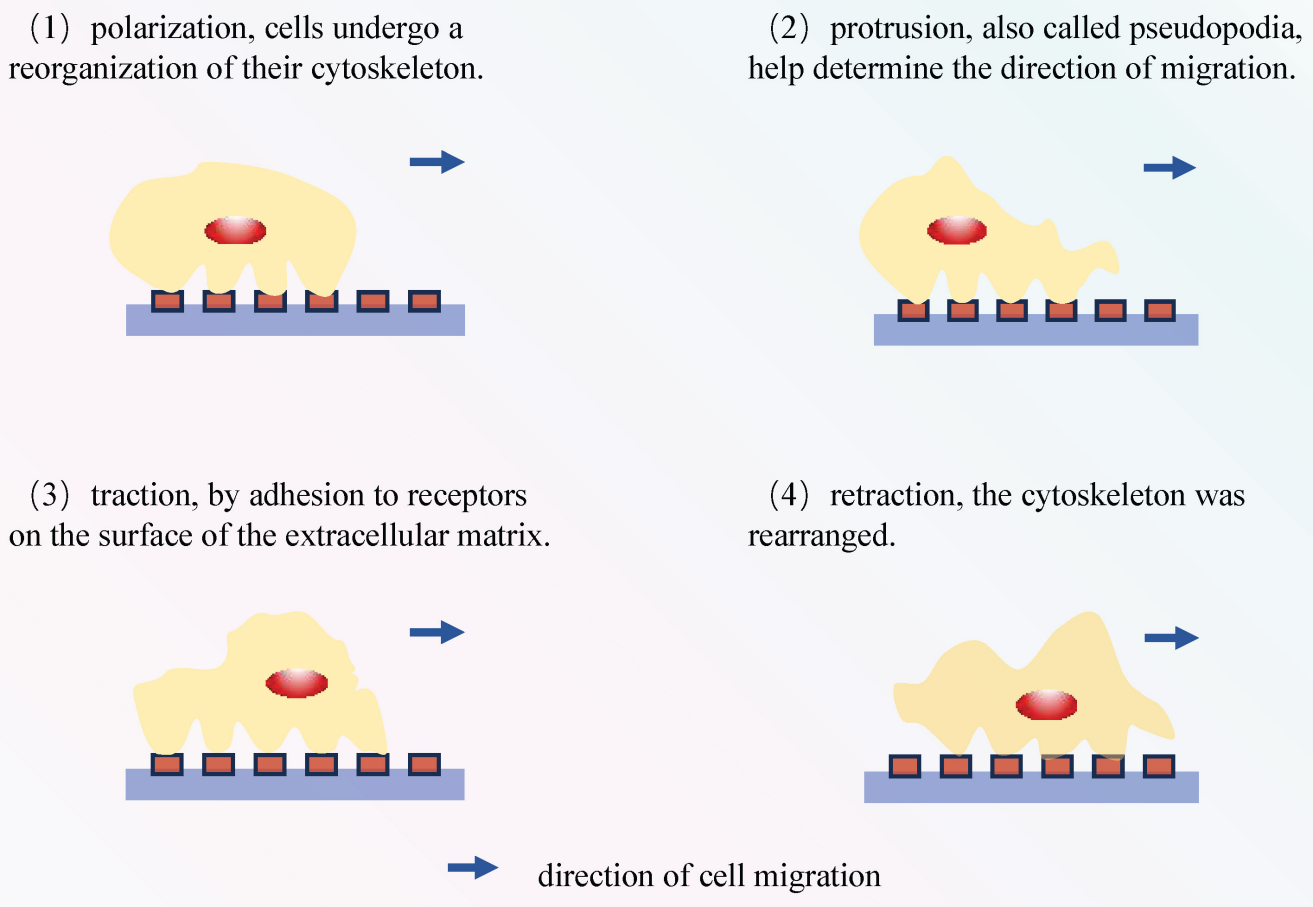
Cell migration process: (1) These cytoskeletal proteins aid in maintaining cell shape and supporting migration. (2) These pseudopodia are supported by the cell's cytoskeleton and assist the cell in extending in a specific direction. (3) Cells interact with the surrounding environment by binding to receptors in the extracellular matrix, allowing them to adhere to the surface. Subsequently, they need to detach by breaking these connections in order to move. (4) Once adhered to a specific location, cells move forward through the reorganization of their cytoskeleton. The above steps were repeated until the cells reached their destination.

The process of cell migration involves sequential events, including polarization, protrusion, traction, and retraction (**Figure 4**) 72, 73. In migratory cells, AQP1 displays polarization towards to the leading edge and is closely associated with membrane protrusions, underscoring its substantial involvement in forward cellular migration (**Figure 3**) 74, 75. Moreover, AQPs facilitate cell migration, signifying a ubiquitous phenomenon that extends beyond specific AQPs and cell types^76^. The excessive migration of immune cells may result in the release of proteases and reactive oxygen species, thereby exacerbating tissue damage^77^. In Rump's study, an increase in bacterial content was observed in wild-type mice, along with a reduction in the expression of NF-κB and mucus secretion following infection, comparable to what was noted in AQP5^-/-^ mice. The diminished expression of AQP5 results in a reduction in immune cell migration, subsequently lowering the 30-day mortality rate in patients with ARDS caused by sepsis. Moreover, an elevation in the C allele content of AQP5 gene-1364A/C promoter SNPs leads to a decrease in the expression of AQP5, thereby contributing to reduced mortality^78^, ^79^. The AQP5 protein not only facilitates cellular migration but also exerts an inhibitory effect on apoptosis in various subtypes of lung cancer. The expression of AQP5 is downregulated in LPS-induced ARDS, and the induction of cell damage by LPS may be associated with an upregulation in apoptosis (**Table 3**)^80^. Therefore, the decrease in AQP5 expression may also be associated with the increase in apoptosis. Mice treated with the tumor cytokine TNF-α, a pro-inflammatory cytokine, exhibited a concentration- and time-dependent reduction in both AQP5 mRNA and protein expression within lung epithelial cells^81^, ^82^. Activation of the p55 TNF-α receptor (TNFR1) using agonist antibodies resulted in a reduction in AQP5 expression^83^, ^84^. Furthermore, inhibition of NF-κB transactivation can mitigate the impact of TNF-α on AQP5 expression^83^. The aforementioned observation suggests that TNF-α exerts a suppressive effect on the expression of AQP5, with TNFR1 and NF-κB serving as indispensable factors (**Figure 3**).

### Sustained inflation of lung and ulinastatin

The pathogenesis of ARDS is closely associated with AQPs, and leveraging this connection makes valuable contributions to clinical practice. Dexamethasone can increase the expression of AQP1 in pulmonary capillary endothelial cells, which may be related to the glucocorticoid response element (GRE) in the AQP1 promoter^85^, ^86^. However, as AQP1 is not strongly implicated in the development of pulmonary edema in ARDS, it is believed that dexamethasone reduces pulmonary edema by mitigating lung inflammation. Sustained inflation (SI) of the lung has been shown to improve oxygenation, lung compliance, and airway resistance in patients with ARDS. This may be attributed to SI's ability to reduce cytokine secretion and subsequent local inflammation and injury, increase the expression of AQP1 and AQP5, as well as promote the clearance of alveolar space and pulmonary interstitial edema fluid^87^. Ulinastatin (UTI) can alleviate the symptoms of ARDS, as it has been demonstrated to improve hemodynamic stability, effectively inhibit the systemic inflammatory cascade, enhance alveolar capillary permeability and transmembrane fluid transport, promote pulmonary gas exchange by upregulating the expression of AQPs, and suppress pulmonary edema^88^.

## AQPs and non-small cell lung cancer

### AQPs dynamics in tumor angiogenesis

The occurrence and progression of tumors intricately attribute to tumor angiogenesis, as well as the proliferation, migration, invasion, apoptosis, and cell cycle regulation of tumor cells^89^. The abundance of tumor blood vessels facilitates the efficient delivery of a large amount of nutrients to the tumor^90^, ^91^. An investigation has verified that AQP1 deficiency affects angiogenesis through subcutaneous Matrigel implantation in mice and partially by adding angiogenic factors such as bFGF and vascular endothelial growth factor (VEGF) 92. The experiments revealed that endothelial cells expressing AQP1 exhibited inherent disparities, which served as the fundamental cause of impaired angiogenesis resulting from AQP1 defects. This observation was also substantiated in additional related experiments^93^, ^94^. In a separate study conducted on a melanoma mouse model, there was an observable elevation in the expression levels of HIF-1α, high-molecular weight DNA fragmentation and caspase-3 (CASP3) within the AQP1 siRNA-treated tumour cells in comparison to the control group. This implies that the silencing of AQP1 may facilitate the formation of a hypoxic environment, thereby influencing tumour cells towards apoptosis^95^. Additionally, the expression of AQP1 was found to be positively correlated with the degree of VEGF expression during the development of endometrial adenocarcinoma^96^.

How does AQP1 impact the process of non-small cell lung cancer (NSCLC) angiogenesis? Under hypoxic conditions, both the mRNA and protein expression levels of AQP1 were markedly increased (**Table 4**), and no alteration in its expression was noted upon the inhibition of VEGF signal transduction. In the presence of typical hypoxia-inducing molecules such as VEGF and erythropoietin (EPO) 97, their time course for gene expression deviates from that of HIF-1, which orchestrates the expression of VEGF and EPO genes during hypoxic conditions^98^, ^99^. The peak expression level of AQP1 mRNA occurs later than those of HIF-1, VEGF, and EPO^100^. Consequently, we propose that hypoxia-induced angiogenesis mediated by AQP1 might be independent of HIF-1 and VEGF. AQP1 may be directly regulated by hypoxia or other factors^92^. In the acute radiation proctitis (ARP) mouse model, the PI3K/AKT pathway can affect NF-Kβ, which in turn promotes VEGF expression and angiogenesis. Treatment of ARP mice with dexamethasone (DXM) combined with gentamicin (GM) enema reduced VEGF and AQP1 expression, resulting in inhibition of angiogenesis. However, the reduction of AQP1 expression by DXM and GM did not involve the PI3K/AKT pathway^101^. The elevated expression of AQP1 serves to alleviate cellular hypoxia resulting from high glucose concentrations^102^. However, the findings from other studies suggest that AQP1 may enhance cytoplasmic hypoxia formation by facilitating oxygen transport across the plasma membrane. This, in turn, could promote the synthesis of HIFα- and oxygen-dependent genes (TH, PGK1, and VEGF), thereby influencing angiogenesis^103^, ^104^.

In addition to AQP1, AQP3 may also play a role in tumor angiogenesis. AQP3 knockdown inhibits tumor angiogenesis by reducing CD31 immunostaining, HIF-2α expression, and VEGF expression^105^. The same conclusion was reached in a diabetic pig burn healing experiment. AQP3 was upregulated in porcine burns and correlated positively with the density of angiogenesis^106^. Therefore, it is hypothesized that elevated expression of AQP1 and AQP3 may facilitate tumor cell formation (**Figure 3**). However, the exact mechanism is still unknown.

### Role of AQPs in tumor proliferation and migration

AQP3, a representative aquaporin facilitating the transport of water, glycerol, and urea, plays a crucial role in preserving fluid homeostasis within normal tissues^107^, ^108^. In previous studies, it was demonstrated that miRNAs can affect cell proliferation and migration by influencing the expression of AQP3^109^, ^110^. The elevated expression of AQP3 is significantly correlated with disease progression and an unfavorable prognosis in lung adenocarcinoma (LUAD), playing a pivotal role in the early stages of LUAD (**Table 4**)^9^. In the absence of AQP3, there is a reduction in glycerol levels and its metabolites, including glycerol-3-phosphate and ATP, occurs within epidermal cells. This deficiency hampers the MAP kinase signaling pathway, while leaving mitochondrial function unaffected. Furthermore, the absence of AQP3 impedes tumor promoter-induced cell proliferation. Nevertheless, the supplementation of glycerol effectively reinstates both cell proliferation and ATP content in the absence of AQP3, thereby restoring cellular functionality^107^, ^111^, ^112^. The wound healing process is characterized by the migration and proliferation of epidermal basal keratinocytes, which are distinguished by a robust expression of the water/glycerol transporter AQP3^113^. In the absence of AQP3, the permeability of the cell membrance to water and glycerol is reduced by more than twofold, which results in a decelerated rate of wound healing^114^. Hence, the glycerol transport facilitated by AQP3 is a crucial supplier of ATP necessary for cellular proliferation.

The proliferation and invasion of NSCLC cells are inhibited by miR-874 through the regulation of the PI3K/AKT signaling pathway and epithelial-mesenchymal transition (EMT), with AQP3 being a target. Conversely, the up-regulation of AQP3 has been observed to promote cell proliferation, thereby counteracting the inhibitory effect of the miR-874 mimic on cell proliferation^115^. Nevertheless, there is presently insufficient direct evidence to substantiate the inhibitory effect of miR-874 on AQP3 expression for the regulation of the PI3K/AKT signaling pathway. In experiments conducted by Y-C Kao and colleagues, the effects of hydrostatic pressure (HP) on cells were investigated. The HP was applied to the cells in order to simulate the interstitial fluid pressure (IFP) of tumours. The results demonstrated that, in an environment of high HP, the degree of p-Caveolin-1 on the cell membrane can affect the phosphorylation of the signalling pathway AKT/ERK. This, in turn, promotes the expression of AQP3 and the proliferation and migration of tumour cells^116^. To some extent, this can also explain the association between AQP3 and the PI3K/AKT signalling pathway.

Moreover, it was postulated that AQP1 also played a role in cellular proliferation. Changes in the expression of Matrix metalloproteinase (MMP)-2, MMP-9, TGF-β, and epidermal growth factor receptor (EGFR) in cells were identified to be linked to the migratory and metastatic capabilities of lung cancer cell lines^117^, ^118^. MMPs is responsible for the breakdown of the extracellular matrix and the subsequent release of associated angiogenic factors^119^. Silencing AQP1 through siRNA significantly decreased the expression levels of MMPs in lung cancer cells with varying AQP1 expression levels. Consequently, this process influences the migration of tumour cells. The reduction in MMP-2/MMP-9 expression displayed a dose-dependent response to AQP1-siRNA, while the downregulation of the AQP1 gene did not significantly affect TGF-β and EGFR. Moreover, RNA interference-mediated silencing of AQP1 was observed attenuate the proliferation and metastasis of lung cancer cells. In conclusion, it can be proposed that AQP1 may promote the proliferation and migration of lung cancer cells in a manner dependent on MMP-2 and MMP-9^120^. The results suggest a positive correlation between the overexpression of AQP1 and AQP3 and the heightened proliferation and migration capabilities of tumor cells (**Figure 3**). In addition, AQP1 has been shown to interact with β-protein, a bifunctional protein that can activate both c-Myc and cyclin D1, and affect the ability of cells to proliferate and migrate, in a variety of diseases^121^. In experiments using A549 cells to construct an AQP1 overexpression model, increased levels of total β-catenin protein but significantly lower levels of phosphorylated β-catenin were observed, as well as an increased proliferation and migration ability of the cells^122^. The precise mechanism remains elusive, although in breast cancer, AQP1 and glycogen synthase kinase-3β (GSK3β) were observed to interact competitively with the 12 armadillo repeats of β-catenin, subsequently inhibiting β-catenin degradation and resulting in β-catenin accumulation in the cytoplasm and nuclear translocation^123^. This may provide some indication as to the direction of our subsequent research.

### AQPs in tumor invasiveness

The primary cause of mortality for cancer patients attributes to tumor metastases^124^. The metastatic cascade of tumor cells reprensents a complex process, initiated by the invasion of adjacent tissues at the primary site, followed by entry into the circulatory system, and eventual penetration into distant organs^125^. Tumor cells that able to survive within these distant organs continue to proliferate, thereby leading to the formation of secondary tumors^125^. The facilitation of cell migration, a phenomenon commonly regulated by AQPs, contributes to the heightened local aggressiveness of tumors. The expression of AQPs in tumor cells is associated with increased metastatic potential, which may be attributed to their ability to facilitate the passage of tumor cells through the microvascular endothelial barrier (**Table 4**)^126^. It is not guaranteed that tumor cells will survive in distant organs upon entering the circulatory system is not assured. It has been demonstrated that apoptosis, which functions as a protective mechanism, may impede the extravasation of malignant tumor cells^125^, ^127^. The formation of finger-like aggregates of tumor cells represents a pivotal stage subsequent to the commencement of cell migration and subsequent entry into the circulatory system. Utilizing 3D culture technology, human NSCLC A549 cells were observed to cluster together and adhere to the actin cytoskeleton in a finger-like manner. Deletion of the AQP3 gene hampers actomyosin cytoskeletal reorganization by impacting caspase 3 activation, consequently impairing the formation of multicellular aggregates in 3D culture^128^.

Recently, an innovative concept has emerged, suggesting that dormant tumor stem cells may play a pivotal role in tumor activation^129^. GSK-3β modifies specific sites on β-catenin, leading to cellular inactivation through ubiquitination^130^. The increased expression of AQP3 in tumor stem cells may lead to the downregulation of genes linked to the Wnt/GSK-3β/β-catenin pathway, specifically GSK-3β and β-catenin. This downregulation inhibits apoptosis in tumor stem cells, thereby augmenting the invasive potential of tumor cells. In a recent study, it was proposed that phosphorylation of AQP5 at Ser156, followed by its interaction with c-Src, could enhance the invasive capacity of tumor cells^131^. Therefore, the elevation of AQP3 and AQP5 expression proves advantageous in augmenting the aggressiveness of tumor cells (**Figure 3**).

### Prognostic significance of AQP4 in NSCLC

The diminished expression of AQP4 on the membrane of tumor tissues is a common observation, with a parallel reduction in expression observed in non-tumor tissues (**Table 4**)^132^, ^133^. Furthermore, AQP4 expression has been detected in NSCLC, particularly in well-differentiated adenocarcinomas. Elevated AQP4 levels in NSCLC are linked to a more favorable prognosis, which may be attributed to its positive correlation with the normal physiological function of the lung. However, the exact mechanism by which this association is established remains unclear^134^.

### AQPs in targeted tumor therapy and early screening

The interplay between AQPs and NSCLC is intricate, yet it is firmly established that elevated AQPs expression may facilitate the development and progression of NSCLC. Moreover, there is potential for the development of AQPs inhibitors that could be used to regulate tumor angiogenesis, proliferative capacities, and invasiveness. Consequently, AQPs hold promise as potential indicators for prognosis assessments and evaluations of distant metastasis in NSCLC.8 Currently, there is a limited body of literature on the utilization of AQPs inhibitors in the treatment of NSCLC. Consequently, researchers are confronted with a substantial task ahead, necessitating a comprehensive understanding of the optimal application of AQPs inhibitors for the treatment of NSCLC.

## Limitations

The review of AQPs in association with respiratory diseases is confronted with certain limitations. Primarily, a significant proportion of experiments have been conducted on mice, posing challenges in extrapolating findings to potential species-specific differences. Considering animals with more extended gestation periods, such as sheep, might offer a more relevant model for testing. Moreover, the restricted sample size of human tissues often fails to dequately address confounding variables like race and geographic location. Although the association between AQPs and respiratory diseases can be inferred through hypothesis verification, cross-validation, and other methodologies, direct validation of this mechanism remains elusive.

## Conclusion

AQPs possess the potential to influence the progression of respiratory diseases by modulating specific signaling pathways, particularly evident in NSCLC. Their roles extend to facilitating transmembrane transport of water and other small molecules, regulating cellular migration, and modulating inflammatory responses. In NSCLC, the overexpression of AQPs is significantly associated with an increased likelihood of tumor progression. The elevated expression of AQP1 and AQP3 not only stimulates tumor angiogenesis but also facilitates tumor proliferation and migration. Furthermore, the relationship between AQP3 and AQP5 and tumor invasiveness should not be overlooked. The intimate connection between AQPs and the pathogenesis of respiratory diseases underscores the need for a comprehensive exploration of their specific mechanisms. Such investigations hold promise for providing valuable insights into clinical diagnosis and the development of effective treatment strategies. Furthermore, changes in the expression of AQPs in pulmonary tissues could be served as an indicator for assessing the effectiveness of novel therapeutic interventions. However, a thorough understanding of the underlying mechanisms of these diseases demands persistent research efforts.

## Figures and Tables

**Table 1:** The expression changes and direct effects of AQPs in COPD

AQP subtype	Expression in COPD	Direct effects
AQP1	No change	No
AQP3	No change	No
AQP4	No change	No
AQP5	Down	It is accompanied by an increase in MUC5AC expression, which promotes mucus secretion

**Table 2:** The expression changes and direct effects of AQPs in Asthma

AQP subtype	Expression in Asthma	Direct effects
AQP1	Up in the small airways, down in the alveolus	Unknown
AQP3	Up	Facilitates the absorption of H_2_O_2_ into the cells
AQP4	Up	Unknown
AQP5	Up in the small airways, down in the alveolus	Unknown

**Table 3:** The expression changes and direct effects of AQPs in ARDS

AQP subtype	Expression in ARDS	Direct effects
AQP1	Up	immune cell migration
AQP3	No change	No
AQP4	No change	No
AQP5	Down	Increase apoptosis

**Table 4:** The expression changes and direct effects of AQPs in NSCLC

AQP subtype	Expression in NSCLC	Direct effects
AQP1	Up	Reduces intracellular oxygen levels and promotes MMP-2 and MMP-9 expression
AQP3	Up	Glycerol entry into cells, leading to ATP generation, caspase 3 activation, and Wnt/GSK-3β/β-catenin
AQP4	Up	Unknown
AQP5	Up	Ser 156 phosphorylation
